# Dry needling and exercise for chronic whiplash - a randomised controlled trial

**DOI:** 10.1186/1471-2474-10-160

**Published:** 2009-12-18

**Authors:** Michele Sterling, Stephanie Valentin, Bill Vicenzino, Tina Souvlis, Luke B Connelly

**Affiliations:** 1 Centre of National Research on Disability and Rehabilitation Medicine, The University of Queensland, Brisbane, Australia; 2 CCRE: Spinal Injury, Pain and Health, Division of Physiotherapy, The University of Queensland, Brisbane, Australia; 3 Australian Centre for Economic Research on Health (ACERH), The University of Queensland, Brisbane, Australia; 4 School of Economics, The University of Queensland, Brisbane, Australia

## Abstract

**Background:**

Chronic whiplash is a common and costly problem. Sensory hypersensitivity is a feature of chronic whiplash that is associated with poor responsiveness to physical treatments such as exercise. Modalities such as dry-needling have shown some capacity to modulate sensory hypersensitivity, suggesting that when combined with advice and exercise, such an approach may be more effective in the management of chronic whiplash. The primary aim of this project is to investigate the effectiveness of dry-needling, advice and exercise for chronic whiplash.

**Method/Design:**

A double-blind randomised controlled trial will be conducted. 120 participants with chronic whiplash, grade II will be randomised to receive either 1) dry-needling, advice and exercise or 2) sham dry-needling, advice and exercise. All participants will receive an educational booklet on whiplash. Participants who are randomised to Group 1 will receive 6 treatments of combined dry-needling and exercise delivered in the first 3 weeks of the 6 week program, and 4 treatments of exercise only in the last 3 weeks of the program. Participants randomised to Group 2 will receive an identical protocol, except that a sham dry-needling technique will be used instead of dry-needling. The primary outcome measures are the Neck Disability Index (NDI) and participants' perceived recovery. Outcomes will be measured at 6, 12, 24 and 52 weeks after randomization by an assessor who is blind to the group allocation of the participants. In parallel, an economic analysis will be conducted.

**Discussion:**

This trial will utilise high quality trial methodologies in accordance with CONSORT guidelines. The successful completion of this trial will provide evidence of the effectiveness and cost-effectiveness of a combined treatment approach for the management of chronic whiplash.

**Trial registration:**

ACTRN12609000470291

## Background

Persistent chronic pain following whiplash injury as a result of a motor vehicle crash is a common and costly problem. In Queensland, Australia, the financial costs related to whiplash injury are substantial and exceeded $500 million from 1994-2001 [1]. The maintenance of activity and exercise is recommended for the management of whiplash [2,3]. However, trials of treatment approaches including exercise and activity for chronic whiplash have demonstrated only modest effects on pain and disability levels [4,5]. One reason for these modest effects may be due to the heterogeneous nature of the whiplash condition. Many people with chronic whiplash have a complex clinical presentation with marked sensory disturbance (widespread hyperalgesia, sympathetic nervous system dysfunction) indicative of central nervous system hyperexcitability [6-9]. The presence of these factors is associated with higher levels of pain and disability and poor functional recovery [10,11]. Our data also indicate that patients with these features show a poor response to an exercise-based intervention approach [5].

Modalities such as dry needling and acupuncture are commonly used in the treatment of musculoskeletal pain conditions [12]. With respect to neck pain of all types and not specifically whiplash, a recent systematic review reported moderate evidence for pain relieving effects of these approaches [13]. Furthermore, these modalities can have a modulatory effect on hyperalgesia [14] and they are effective in alleviating symptoms of fibromyalgia [12], a condition that also features characteristics of central hyperexcitability [15]. Together these findings suggest that needling techniques may be effective in the management of chronic whiplash pain, but the use of such interventions in this condition has never been investigated. A recent Cochrane review concluded that dry-needling, added to other conventional therapies such as exercise, is more effective at relieving pain than conventional therapies alone in non-specific low back pain [16]. This combined approach to management has never been investigated in whiplash. Therefore the primary aim of this project is to investigate the effectiveness of dry-needling, advice and exercise compared with sham dry-needling, advice and exercise in chronic whiplash. Effectiveness will be measured by reductions in pain and disability levels and improvements in patients' impressions of overall recovery and quality of life. The secondary aim is to conduct an economic evaluation of the dry-needling, advice and exercise approach.

## Method/Design

### Design

**Figure 1:**
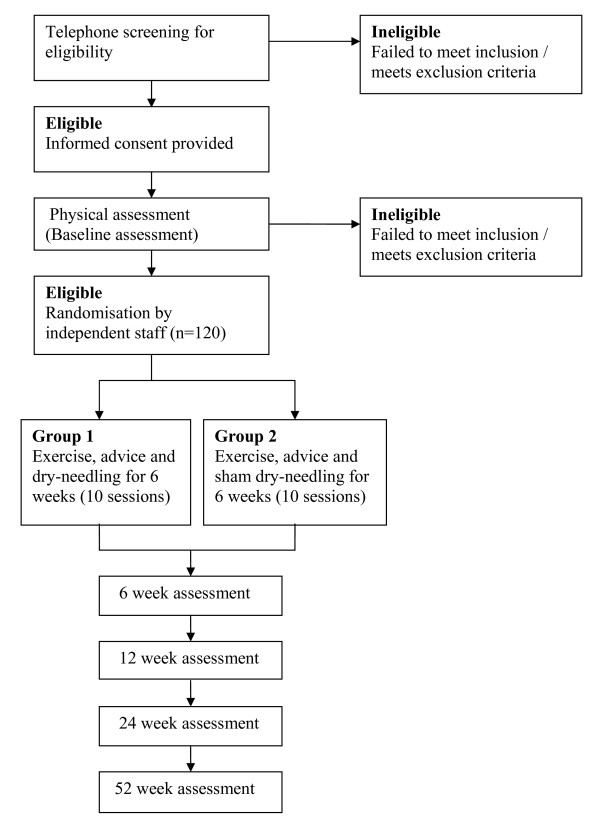
**Flow-diagram of trial protocol**.

Double-blind randomised controlled trial. Ethics approval has been granted from the Human Research Ethics Committee of the University of Qld (2008002314). Figure 1 shows the flow of participants through the study.

### Setting

Participants will be assessed at a research laboratory at The University of Queensland. The interventions will be provided at community physiotherapy practices in Brisbane and Ipswich, Queensland.

### Participants

A total of 120 participants with chronic whiplash will be recruited. Inclusion criteria are Whiplash Grade II (no fracture or dislocation and no clinical neurological deficit on clinical examination) [17] of at least 3 months but less than 2 years in duration; moderate pain and disability (Neck Disability Index minimum of 28/100); and presence of sensory hypersensitivity, defined as 2 out of a possible 3 of the following: cold pain threshold (> 15°C, males and females), pressure pain thresholds over the cervical spine (females < 185 kPa, Males < 210 kPa), and pressure pain thresholds over the median nerve (females < 210 kPa, Males < 250 kPa) *(6)*. Participants must also be naive to dry-needling and acupuncture techniques and must not be receiving any other treatment for their whiplash symptoms at the time of recruitment. Exclusion criteria are known or suspected serious spinal pathology (e.g. metastatic, inflammatory or infective diseases of the spine); spinal surgery in the past 12 months; diagnosis with a mental health condition; confirmed fracture or dislocation at time of injury (Whiplash Grade IV); and nerve root compromise defined as at least 2 of the following signs: weakness, reflex changes, or sensory loss (Whiplash Grade III)[17]. Participants will be recruited from local medical practices and via electronic and print media advertising.

### Intervention

All participants will be provided with the patient educational booklet 'Whiplash injury recovery: A self management guide' published by the Motor Accident Insurance Commission (Queensland)[18], which provides information about whiplash, advice on symptom management and illustrates a simple exercise program. Participants will be randomised to one of two groups. Participants in Group 1 will receive 6 treatments of combined dry-needling and exercise delivered in the first 3 weeks of the 6 week program, and 4 treatments of exercise only in the last 3 weeks of the program. Participants in Group 2 will receive an identical protocol, except that a sham dry-needling technique is used instead of dry-needling.

Sham dry-needling uses needles which cause a pricking sensation when pushed against the skin. The needle disappears into the handle with increased pressure. This mechanism invokes a similar sensation to that produced by dry-needling, which includes the non-specific effects of clinician -therapist contact and explanation, as well as the perception that the needle is entering the skin, although sham dry-needling is proposed to have less physiological effect than true needling[19]. Needling sites will be dependent on findings by the physiotherapist following assessment for the presence of hyperalgesia on palpation. Only posterior muscles of the cervical spine and upper thoracic spine will be treated whilst the participant is lying prone, to ensure the participant remains blinded to the needling group allocation. Examples of posterior muscles which may be treated are the trapezius, levator scapulae, splenius capitus, semispinalis and spinalis capitis muscles.

The 6-week exercise program will be carried out under supervision from the physiotherapist and will comprise specific exercises to improve the movement and control of the neck and shoulder girdles (see Table 1). The exercises will be tailored by the physiotherapist for each individual participant, are of a low load nature and are designed to be pain free. At the same time, the physiotherapist will guide the subject's return to normal activities. This programme is similar to those utilised by physiotherapists in clinical practice and has been used in previous studies[5].

**Table 1:** List of Exercises

Exercise	Description
1. Flexors of the cranio-cervical spine	Re-education of cranio-cervical flexion movement Training holding capacity of the neck flexors Retraining eccentric control of the cranio-cervical flexors in upright postures

2. Extensors of the cranio-cervical spine	Re-education of cranio-cervical extension movement Training holding capacity of the neck extensors

3. Re-education of neutral posture	Re-education of neutral spine in sitting, including lumbo-pelvic, thoracic and cervical spine neutral Progression to other functional postures (patient specific)

4. Retraining of the scapular muscles	Retraining scapular orientation in neutral posture Training endurance capacity of the scapular stabilizers Retraining dynamic scapular control with arm movement and load

5. Co-contraction of the neck flexors and extensors	Facilitated with rotation, using self-resisted isometric rotation in either supine, or in correct upright sitting posture

6. Strength training of the flexor synergy	Head lift in supine, preceded with cranio-cervical flexion followed by cervical flexion to just lift the head from the supporting surface. Graded reduction in pillow height to flat surface

### Outcome Measures

Age, gender, time since injury, compensation status, previous treatments and medication usage for the whiplash condition will be obtained at baseline. The assessor administering the outcomes will be blind to the group allocation of the participant. Outcome measures will be assessed at baseline, 6, 12, 24 and 52 weeks after randomization.

### Primary outcome measures

1. Neck-specific measure of disability (Neck Disability Index)[20]

2. Patient's global impression of recovery (-5 to +5 scale) [21]

### Secondary outcome measures

3. Average pain intensity over last week (Visual Analogue Scale)[21]

4. Average pain intensity over last 24 hours (Visual Analogue Scale)[21]

5. Patient-generated measure of disability (Patient-Specific Functional Scale)[22]

6. Whiplash-specific measure of disability (Whiplash Disability Questionnaire) [23]

7. Generic measure of health status (SF-36) [24]

8. Measures of physical impairment (Cervical range of movement, pressure pain threshold, cold pain threshold) [25]

9. Impact of events scale (IES) [25]

10. Pain Catastrophising Scale (PCS)

11. Posttraumatic stress Diagnostic Scale (PDS)

12. Self report Leeds Assessment of Neuropathic Symptoms and Sign (S-LANSS Pain Score)[25]

### Adverse Events

All adverse effects, defined as any negative or unwanted reactions to the interventions will be recorded. These will include increased pain (greater than 2 points the VAS scale for greater than 1 week), skin infection, skin irritation or any other reported physical discomfort. An adverse effects committee (chaired by MS) will manage any adverse reactions as they occur. Adverse effects of treatment will be identified using open-ended questioning at the 6 week follow-up assessment.

### Randomisation

Participants will be randomly allocated to groups by concealed allocation. A statistician who is independent of the study will be responsible for generating the computerised randomisation schedule. An independent research assistant will administer the schedule and perform all communication between participants and treating practitioners.

### Sample size calculations

The NDI was used to determine sample size, as previous clinical trials have shown this variable to have a smaller effect size than the measure of perceived recovery [5]. The minimal clinically important change for the NDI is 7 points [26] with a conservative standard deviation of 18 based on previous work[5,27]. Based on 80% power at *p *= 0.05, 54 participants will be required in each group. Allowing for a 10% loss to follow up, a total of 60 participants will be required in each group, with 120 participants in total.

### Data management and analysis

All analyses will be conducted on an intention to treat basis. The outcomes measured at 6, 12, 24 and 52 weeks will be analysed using linear mixed and logistic regression models that will include their respective baseline scores as a covariate, subjects as a random effect and treatment conditions as fixed factors. Age, gender, duration of condition and IES scores will be included as covariates in the analysis. Regression diagnostics will be used to check for normality of the measures and homogeneity of variance as appropriate. Alpha will be set at 0.01.

### Data integrity

The integrity of trial data will be monitored by regularly scrutinising data sheets for omissions and errors. Data will be double-entered and the source of any inconsistencies will be explored and resolved.

## Discussion

Although many people experience rapid recovery from pain and disability associated with whiplash within the first three months, a significant proportion can have ongoing mild to moderate pain and disability[28]. It has been shown that the presence of widespread sensory hypersensitivity indicative of augmented pain processing mechanisms is associated with higher levels of pain, disability and poor functional recovery following whiplash injury [29]. Chronic patients with these features have been shown to have a poor response to an exercise-based intervention approach[5]. In order to enhance the treatment outcomes of an exercise intervention, an additional modality or technique could be used in conjunction with an exercise programme to address the sensory hypersensitivity of whiplash. Modalities such as dry needling and acupuncture are commonly used in the treatment of musculoskeletal pain conditions[12], although the benefit of dry needling for the treatment of whiplash is not yet known. Therefore, this trial aims to investigate whether the addition of dry needling to an exercise programme will reduce pain and disability of chronic whiplash.

The exercise programme received by all participants in the trial includes specific exercises to re-educate cervical and scapular muscle control endurance and strength and are of low-load in nature. These exercises are tailored to the ability of the participants by the trial physiotherapists. This type of exercise programme has previously shown modest effects in the treatment of chronic whiplash by reducing pain and disability [4,5] and was therefore the programme of choice for this trial.

A sham dry needling intervention will be used as a in the comparison group to assess the effects of dry-needling on whiplash. Sham dry needling invokes a similar sensation to that produced by true dry-needling, without the needle entering the skin. This technique is proposed to have a lesser physiological effect than true needling [19]. Application of the sham and true dry needles are to the posterior cervical and thoracic spine whilst the participant is prone, which will allow participant blinding to their group allocation. A blinded independent trial assessor will conduct participant assessments. The methodology of this double-blind randomised controlled trial meets the CONSORT statement and guidelines [30].

An economic evaluation of the dry-needling, advice and graded exercise approach will also be conducted as a secondary aim in this trial. As the economic cost of whiplash injury is extensive, developing effective treatment programmes which minimise pain, disability and resultant economic cost is desirable.

It is hypothesised that the dry needling approach will decrease the pain and disability of chronic whiplash by reducing the sensory hypersensitivity and facilitate the effects of the exercise program. The study outcomes will facilitate the development of effective treatment plans for chronic pain and disability following a whiplash injury and have the potential to reduce the economic burden of this condition.

## Competing interests

The authors declare that they have no competing interests.

## Authors' contributions

MS, BV, TS and LC participated in the conception and design of this trial and are chief investigators on the NHMRC grant # 569832. MS, SV, BV, TS and LC were responsible for writing this manuscript. All authors read and approved the final manuscript.

## Pre-publication history

The pre-publication history for this paper can be accessed here:

http://www.biomedcentral.com/1471-2474/10/160/prepub

## References

[B1] MAIC Annual report 2003-4 2004 Motor Accidents Insurance Commission

[B2] Scholten-Peeters G Bekkering G Verhagen A Windt D Van der Lanser K Hendricks E Oostendorp R Clinical practice guideline for the physiotherapy of outpatients with whiplash associated disorders Spine 2002 27 4 412 422 10.1097/00007632-200202150-00018 11840109

[B3] MAA Guidelines for the management of whiplash associated disorders 2007 Sydney: Motor accidents authority 16

[B4] Stewart M Maher C Refshauge K Herbert R Bogduk N Nicholas M Randomised controlled trial of exercise for chronic whiplash associated disorders Pain 2007 128 1-2 59 68 10.1016/j.pain.2006.08.030 17029788

[B5] Jull G Sterling M Kenardy J Beller E Does the presence of sensory hypersensitivity influence outcomes of physical rehabilitation for chronic whiplash? - A preliminary RCT Pain 2007 129 2 28 34 10.1016/j.pain.2006.09.030 17218057

[B6] Sterling M Jull G Vicenzino B Kenardy J Sensory hypersensitivity occurs soon after whiplash injury and is associated with poor recovery Pain 2003 104 509 517 10.1016/S0304-3959(03)00078-2 12927623

[B7] Sterling M Kenardy J Jull G Vicenzino B The development of psychological changes following whiplash injury Pain 2003 106 3 481 489 10.1016/j.pain.2003.09.013 14659532

[B8] Raak R Wallin M Thermal thresholds and catastrophising in individuals with chronic pain after whiplash injury Biol Res Nurs 2006 8 2 138 146 10.1177/1099800406291078 17003253

[B9] Kasch H Reduced cold pressor pain tolerance in non-recovered whiplash patients: a 1 year prospective study European Journal of Pain 2005 9 1 561 569 10.1016/j.ejpain.2004.11.011 16139185

[B10] Sterling M Jull G Kenardy J Physical and psychological predictors of outcome following whiplash injury maintain predictive capacity at long term follow up Pain 2006 122 102 108 10.1016/j.pain.2006.01.014 16527397

[B11] Sterling M Jull G Vicenzino B Kenardy J Darnell R Physical and psychological factors predict outcome following whiplash injury Pain 2005 114 141 8 10.1016/j.pain.2004.12.005 15733639

[B12] Ernst E Musculoskeletal conditions and comlplementary/alternative medicine Best practice and research clinical rheumatology 2004 18 4 539 556 10.1016/j.berh.2004.03.005 15301985

[B13] Trinh K Graham N Gross A Goldsmith C Wang E Cameron I Kay T Acupuncture for neck disorders Spine 2007 32 2 236 243 10.1097/01.brs.0000252100.61002.d4 17224820

[B14] Kong J Fufa D Gerber A Rosman I Vangel M Graceley R Gollub R Psychophysical outcomes from a randomised pilot study of manual, electro and sham acupuncture treatment on experimentally induced thermal pain Journal of Pain 2005 6 1 55 64 10.1016/j.jpain.2004.10.005 15629419

[B15] Mclean S Clauw D Abelson J Liberzon I The development of persistent pain and psychological morbidity after motor vehicle collision: integrating the potential role of stress response systems into a biopsychosocial model Psychosomatic Medicine 2005 67 783 790 10.1097/01.psy.0000181276.49204.bb 16204439

[B16] Furlan A van Tulder M Cherkin D Tsukayama H Lao L Koes B Berman B Acupuncture and dry needling for low back pain: an updated systematic review within the framework of the Cochrane Colaboration Spine 2005 30 8 944 963 10.1097/01.brs.0000158941.21571.01 15834340

[B17] Spitzer W Skovron M Salmi L Scientific Monograph of Quebec Task Force on Whiplash Associated Disorders: Redifing 'Whiplash' and its management Spine 1995 20 1 73 7604354

[B18] Jull G Whiplash injury recovery - a self-management guide 2005 http://www.maic.qld.gov.au/forms-publications-stats/pdfs/whiplash-injury-recovery-booklet.pdf

[B19] White P Lewith G Hopwood V Prescott P Pain 2003 106 3 401 409 10.1016/j.pain.2003.08.013 14659523

[B20] Vernon H Moir S The Neck Disability Index: A study of reliability and validity Journal of Manipulative Physiological Therapeutics 1991 14 7 409 415 1834753

[B21] Pengel L Refshauge K Maher C Responsiveness of pain, disability and physical impairment outcomes in patients with low back pain Spine 2004 29 879 883 10.1097/00007632-200404150-00011 15082988

[B22] Westway M TStratford P Binkley J The patient-specific functional scale: validation of its use in persons with neck dysfunction JOSPT 1998 27 5 331 338 9580892 10.2519/jospt.1998.27.5.331

[B23] Willis C Niere K Hoving J Green S O'Leary E Buchbinder R Reproducibility and responsiveness of the Whiplash Disability Questionnaire Pain 2004 110 3 681 688 10.1016/j.pain.2004.05.008 15288409

[B24] Ware J Sherboure C The MOS 36-item short-form health survey (SF36). 1. Conceptual framework and item selection Med Care 1992 30 473 483 10.1097/00005650-199206000-00002 1593914

[B25] Sterling M Kenardy J Physical and psychological aspects of whiplash: Important considerations for primary care assessment Manual Therapy 2008 13 93 102 10.1016/j.math.2007.11.003 18221907

[B26] Cleland J Fritz J Whitman J Palmer J The reliability and construct validity of the neck disability index and patient specific functional scale in patients with cervical rediculopathy Spine 2006 31 5 598 602 10.1097/01.brs.0000201241.90914.22 16508559

[B27] Stewart M Maher C Advice or exercise for chronic whiplash associated disorders? Design of a randomised controlled trial BMC musculoskeletal disorders 2003 4 18 10.1186/1471-2474-4-18 12932301 PMC194710

[B28] Kamper S Rebbeck T Maher C McAuley J Sterling M Course and prognostic factors of whiplash: a systemic review and meta-analysis Pain 2008 3 617 629 10.1016/j.pain.2008.02.019 18407412

[B29] Carroll LJ Holm LW Hogg-Johnston S Course and prognostic factors for neck pain in Whiplash Associated Disorder (WAD): Results of the bone and joint decade 2000-2010 Task Force on neck pain and its associated disorders Journal of Manipulative and Physiological Therapeutics Supplement 2009 32 2 S97 107 10.1016/j.jmpt.2008.11.014 19251080

[B30] Moher D Schultz KF Altman DG The CONSORT statement: revised recommendations for improving the quality of reports of parallel group randomised trials BMC Medical Research Methodology 2001 1 2 11336663 10.1186/1471-2288-1-2 PMC32201

